# Visualization of Whole-Night Sleep EEG From 2-Channel Mobile Recording Device Reveals Distinct Deep Sleep Stages with Differential Electrodermal Activity

**DOI:** 10.3389/fnhum.2016.00605

**Published:** 2016-11-29

**Authors:** Julie A. Onton, Dae Y. Kang, Todd P. Coleman

**Affiliations:** ^1^Institute for Neural Computation, University of California, San DiegoLa Jolla, CA, USA; ^2^Naval Health Research CenterSan Diego, CA, USA; ^3^Department of Bioengineering, University of California, San DiegoLa Jolla, CA, USA

**Keywords:** EEG, sleep, spectral decomposition, electrodermal activity, deep sleep, slow wave sleep, sleep scoring algorithm

## Abstract

Brain activity during sleep is a powerful marker of overall health, but sleep lab testing is prohibitively expensive and only indicated for major sleep disorders. This report demonstrates that mobile 2-channel in-home electroencephalogram (EEG) recording devices provided sufficient information to detect and visualize sleep EEG. Displaying whole-night sleep EEG in a spectral display allowed for quick assessment of general sleep stability, cycle lengths, stage lengths, dominant frequencies and other indices of sleep quality. By visualizing spectral data down to 0.1 Hz, a differentiation emerged between slow-wave sleep with dominant frequency between 0.1–1 Hz or 1–3 Hz, but rarely both. Thus, we present here the new designations, Hi and Lo Deep sleep, according to the frequency range with dominant power. Simultaneously recorded electrodermal activity (EDA) was primarily associated with Lo Deep and very rarely with Hi Deep or any other stage. Therefore, Hi and Lo Deep sleep appear to be physiologically distinct states that may serve unique functions during sleep. We developed an algorithm to classify five stages (Awake, Light, Hi Deep, Lo Deep and rapid eye movement (REM)) using a Hidden Markov Model (HMM), model fitting with the expectation-maximization (EM) algorithm, and estimation of the most likely sleep state sequence by the Viterbi algorithm. The resulting automatically generated sleep hypnogram can help clinicians interpret the spectral display and help researchers computationally quantify sleep stages across participants. In conclusion, this study demonstrates the feasibility of in-home sleep EEG collection, a rapid and informative sleep report format, and novel deep sleep designations accounting for spectral and physiological differences.

## Introduction

Sleep is a requisite aspect of human existence whose most obvious function is to rejuvenate the brain and body on a daily basis. How sleep achieves this result is not completely understood, but over decades, research has revealed many physiological characteristics of sleep, from molecules to behavior, that have initiated very compelling hypotheses (Tononi and Cirelli, 2006). Of course, many of the methods for exploring the physiology are highly invasive and only conducted on animals. However, human participants can be monitored using a variety of devices to learn more about sleep patterns. For example, simple movement measures from a wrist accelerometer can roughly estimate total sleep time in a multi-day recording. Likewise, heart rate and respiration could potentially differentiate between sleep and wake time. However, brain activity that is expressed in the electroencephalogram (EEG) as detected at the scalp, along with eye and muscle movements, gives additional insight into brain mechanisms of sleep, while still remaining non-invasive and safe. Unlike accelerometers, EEG detects not just lack of movement, but the sleep stages visited over the course of the night.

The gold standard for sleep scoring remains visual scoring of polysomnography (PSG), which includes EEG and heart rate, as well as muscle and eye movement data, among other measures (Rechtschaffen and Kales, 1969). Heart rate typically drops with the onset of sleep (Baust and Bohnert, 1969); loss of muscle tone and rapid eye movement (REM) are strong indicators of REM sleep. PSG provides a summary hypnogram of sleep architecture that is useful in sleep diagnosis and yields an inter-rater consistency of about 80% (Danker-Hopfe et al., 2009). However, visual scoring in 30-s increments is limited since the human eye is minimally sensitive to slight variations in frequency. For example, high- vs. low-frequency spindles, or slow waves of variable frequency, can be difficult to detect and quantify. Therefore, it would be useful to include computational analysis to maximize observations that may not be obvious to even the most trained eye of a sleep technician.

Standard sleep scoring guidelines do not differentiate between various frequencies of slow waves, as long as they are below 3 Hz. However, the research literature has distinguished EEG oscillations below and above 1 Hz that are hypothesized to originate in the cortex and thalamus, respectively (Steriade et al., 1993). Despite this differentiation, slow oscillations (<1 Hz) and delta (1–3 Hz) have never been considered separate sleep stages, but rather different facets of the same deep sleep phenomenon. Part of the reason for this may be that early EEG amplifiers often had built in high-pass filters around 0.1 Hz to prevent saturation from drift potentials, so the lowest frequency slow waves may have actually been absent. Modern amplifiers, which are capable of full spectrum recordings that allow for detection and quantification of slow oscillations, should therefore be re-examined using computational tools that can quantify power in this very low range.

Electrodermal activity (EDA), which is traditionally a measure of sympathetic nervous system activation, was first recorded along with overnight PSG in the early 1960’s, showing, to the surprise of most, that sleep was actually not as autonomically quiescent as it might appear behaviorally. In fact, it was shown that EDA was stronger during deep sleep than any other sleep stage or even waking levels (Johnson and Lubin, 1966; Hori et al., 1970). Skin conductance changes are divided into two broad categories; skin conductance responses (SCRs) and skin conductance level (SCL), which describe phasic and tonic shifts in conductance, respectively. In the awake condition, both are associated with increases in arousal (Dawson et al., 2007). During sleep, both SCRs and SCL are elevated predominantly during deep sleep, but these shifts are unlikely to be due to heightened arousal, as they are in waking conditions (Hori et al., 1970). EDA has several separate control mechanisms in the awake animal, but the mechanism for EDA increase during sleep has not been investigated. As such, it is unknown if EDA during sleep is even mechanistically similar to waking SCR and SCL signals. Despite the consistency of the EDA signals during sleep, EDA recordings have somehow not become a standard measure to help identify deep sleep (Sano et al., 2014).

Various automatic sleep scoring algorithms have been proposed over many years, but none have attracted enough attention to replace or even supplement the accepted visual scoring protocol (Rechtschaffen and Kales, 1969; Silber et al., 2007). Various approaches have been proposed, from simple rule-based decision trees (Liang et al., 2012) to supervised classifiers (e.g., support vector machines or neural networks; Pardey et al., 1996; Sousa et al., 2015), and finally unsupervised classifiers such as Hidden Markov Models (HMMs; Flexer et al., 2002, 2005; Pan et al., 2012). Because traditional PSG uses several EEG channels along with electromyogram (EMG) and electrooculogram (EOG) information, many automatic sleep scoring algorithms use much of the same data associated with PSG (Pan et al., 2012). Some have attempted 1 or 2 channel classification schemes in order to move toward a more streamlined approach to sleep assessment (Flexer et al., 2005; Berthomier et al., 2007). Most algorithms use a combination of spectral measures (Pan et al., 2012; Yaghouby and Sunderam, 2015) as inputs to their algorithms, but some use raw data measures (Flexer et al., 2005). The accuracy of the published algorithms falls between 70% and 95% accuracy for at least 1 sleep stage, which is typically either slow-wave sleep or awake. What most methods still lack is a way for clinicians to view an entire night’s sleep as anything other than a final hypnogram, which displays none of the actual data that led to the sleep categorization. One group has introduced a whole-night sleep visualization (Koupparis et al., 2014), but the final display lacks the visual resolution to convey sleep architecture at a glance.

The present study sought to alleviate some of the disadvantages of prior sleep studies and sleep scoring approaches by offering an intuitive display of whole-night sleep architecture as a normalized time-frequency color image, and importantly, as a dot matrix highlighting the relative dominant frequency at each moment. This technique revealed an important distinction within slow wave sleep between dominant power above or below 1 Hz. The display also includes a hypnogram produced by an HMM, which is implemented with expectation-maximization (EM) model-fitting and Viterbi sequence estimation to deduce sleep stages using only a single channel of EEG data from the forehead. We used a mobile 2-channel EEG device for data collection that allows participants to sleep in the comfort of their own homes at a much reduced cost. Using these techniques, sleep EEG activity can be easily and inexpensively collected, assessed and quantified by researchers or clinicians.

## Materials and Methods

### Participants

Participants were recruited from the San Diego area by public advertisement and included a total of 51 participants (26 males, 25 females) who were medication-free and self-reported asymptomatic sleepers. Mean age of participants was 27.8 years (standard deviation [SD] = 6.1; range 19–40). Participants reported no neurologic or psychiatric disorders, and no history of traumatic brain injury. Their mean Pittsburgh Sleep Quality Index (PSQI) score was 2.5 (SD = 1.4). Mean ± SD self-reported sleep quality for all participants on all nights was 7.0 ± 1.7 on a scale of 1–10 (10 = excellent sleep). Coffee drinking up to three cups per day was permitted, but participants were asked to not consume any excess caffeine compared with their normal amount on days of recordings. Participants were also asked to refrain from alcohol consumption on recording days.

Participants reported to the laboratory for consenting, questionnaires, and explanation of how to use the mobile EEG recording device. Once participants had completed three nights of sleep recordings, which could be consecutive or not but generally within 2 weeks, they returned the device and received compensation for their participation.

### Equipment

Each participant received one of two possible EEG recording devices, both of which included four electrode placements: one above each eye on the forehead and one behind each ear on the mastoid. One device used standard electrocardiogram (ECG) electrode wires leading off the pillow to a small Avatar amplifier (EGI, Eugene, OR) placed above or to the side of the pillow (used by 25 subjects). The Avatar amplifier digitized the data at 500 Hz and has no hardware filters. The other device was a headband design with wires leading to a small recording unit which was attached to an over-band on the top of the head (Cognionics, San Diego, CA, USA). The Cognionics amplifier has no hardware filters and digitized the data at 540 Hz (used by 26 subjects). While each device recorded at different gains and therefore different absolute measurement values, raw data from both devices were comparable. When transformed into frequency space, converted to dB and baselined relative to participant-specific mean spectra, data from both devices produced comparable spectrograms and hypnograms. Specifically, absolute and percent time spent in each sleep stage was not significantly different between devices. Therefore data from both devices were merged in the final analysis. One of the goals of this project was to develop a platform that can use any EEG recording device and deliver comparable analysis and visualization as output. EDA was collected using a multifunctional wrist band that also collected acceleration, heart rate and temperature (Empatica, Milano, Italy).

### Procedure

Participants were instructed to rub forehead and mastoids with an alcohol preparation pad and then let the areas dry completely. The standard ECG electrode stickers were applied above each eye and behind both ears. Photographs with proper placement were included with the device. Lead wires were then snapped in place, and the Empatica device was placed on the left wrist like a watch. When the subject was ready to close their eyes to fall asleep, the Empatica device was turned on, followed by the EEG device immediately thereafter so that they were approximately synchronized. When participants woke up in the morning, they turned off both devices. Participants completed a brief sleep journal rating how well they slept, how exhausted they were the night before, and whether anything woke them up in the middle of the night. Each subject repeated this procedure on three separate nights that were expected to be unrestricted by abnormal time constraints and consistent with their normal sleep patterns. While laboratory sleep studies often disregard the first night of recording as an accommodation night, we found no significant differences between the first and third nights in terms of absolute or percent time in each stage, total sleep time, sleep onset latency, or EDA activity in any sleep stage. We did find a significant difference in subjective sleep quality (*p* < 0.02) indicating slightly improved impression of good sleep on the third night compared to the first. However, because none of the objective measures evaluated in this report were correlated with these subjective evaluations, we used all successfully recorded nights from each subject for the subsequent analysis. Similarly, no significant differences in objective sleep measures were detected between nights rated 5 or less out of 10 for sleep quality, as compared to nights rated 6 or higher. Therefore, all successfully recorded nights were included in the subsequent analysis regardless of subjective sleep ratings.

### Data Processing and Visualization

Data were imported into MATLAB (Mathworks, Natick, MA, USA) and stored as EEGLAB datasets. Channels FP1-A2 and FP2-A2 were analyzed independently. The difference channel FP1-FP2 (forehead-forehead, or FF) was also analyzed because it often showed equivalent results for frequencies under ~30 Hz, but showed significant reduction in >30-Hz power that may have been muscle or temporal lobe brain activity detected at the mastoid reference electrode. Eliminating this high frequency activity by using the FF channel decreased false awakening determinations. The difference channel can also enhance spindle power—making light sleep easier to detect—but it also eliminates waking alpha power which is likely projected from a single occipital source canceled in the difference. Occasionally the difference between frontal electrodes can eliminate very low-frequency oscillations that are synchronous at both forehead electrodes, but usually the difference retains all pertinent features of individual channels with sufficient power. Practically speaking, it is useful to look at all channels to gain a fuller picture. However, for the purposes of this report, we will discuss the FF channel because it shows the most reliable hypnograms compared with either FP1 or FP2 referenced to the mastoid.

The sleep scoring algorithm receives information from a single channel of EEG for each calculation. The algorithm transforms the temporal data into frequency power between 0.1–150 Hz for every 0.5-s time step. Spectral decomposition was accomplished by Morlet wavelet analysis, using three cycles at the lowest frequency and 30 cycles at the highest frequency; frequencies in between used evenly distributed numbers of cycles between 3 and 30. Thus, for a given time step, each frequency considers a different amount of time, from 30 s at 0.1 Hz to 0.2 s at 150 Hz. The real portion of the power was extracted by multiplying the complex power with its conjugate. The result was converted to decibels (dB) by the formula 10*log10(power). The spectrogram was smoothed across time with a 40-s moving average for the display spectrogram only. All other displays and computations used the unsmoothed spectrogram. Large artifacts in the raw EEG data were assumed to be movement and tagged in order to temporarily remove them for calculation of the spectral baseline. Artifact was defined by taking the maximum absolute value of each 30-s stretch of EEG data minus its mean and then marking all windows that exceed 5 SDs from all other windows of data across the night. The 30-s window was chosen because it is the longest window used in the spectral analysis, and 5 SDs was chosen as a value that was well above normal variations in sleep EEG so as to only tag the most extreme outlier activity in the data. The average power spectrum across the entire night was then calculated from all time points except these large artifacts. The baseline spectrum was subtracted from the raw spectrogram to improve visualization of relative spectral changes over the night. Time windows that were tagged as large artifact were not deleted or cleaned for calculations or visualization; they were detected solely to ignore when calculating the average power spectrum since large artifact can skew the average and cause misleading relative spectrograms. These artifactual time points are denoted in the sleep report to aid interpretation and specify when spectral time points were ignored for calculation of the spectral baseline. Line noise was masked in specified frequencies (usually 50–70 Hz but sometimes 110–130 Hz was additionally required) by replacing line frequency values with the average of the two frequency bins below and the two above the noise frequency bins. The line noise frequencies were not used for hypnogram determinations since the line noise masking only affects the displays.

The dominant frequency display was calculated from the unsmoothed, baselined spectrogram to enhance visual detection of dominant frequencies, which is especially helpful for low-spectral power states such as REM sleep. The dominant frequency is determined by finding, at each time point, the frequency with the maximum relative power from the baselined spectrogram. The result is portrayed in the display as tiny dots corresponding to the frequency with the highest relative power at each time point and whose visual gestalt should immediately convey the general sleep cycle pattern.

### Sleep Scoring Algorithm

To create the input for the HMM algorithm, relative power across the entire night was averaged in five frequency bands, which roughly corresponded to five stages of sleep/wake (Wake: 37–47 Hz; REM: 16–30 Hz; Light: 10.5–16 Hz; Hi Deep: 1–3 Hz; Lo Deep: 0.1–1 Hz). Except for the differentiation between Hi and Lo Deep sleep, these frequency bands were based on conventional sleep scoring as well as other published observations (Silber et al., 2007; Kokkinos et al., 2009; Koupparis et al., 2014).

The relationships between sleep stages and the five EEG frequency features across time intervals was modeled as an HMM (Rabiner and Juang, 1986). In this model, each discrete time unit corresponds to a 30-s epoch, and it is assumed that the participant is in one of the five aforementioned hidden stages of sleep or wake. This discrete time process was modeled as a Markov process, where the transition probabilities are specified by the 5 × 5 Q matrix, where Q(j|i) is the probability of being in stage j at time *t* + 1 given that the participant is in stage i at time *t*. Each 30-s interval of data was transformed into a 5-dimensional observation vector, y, by extracting the average log power of the EEG in each of the five bands in that time window. R(y|j) is the conditional probability of observing the 5-dimensional feature vector, y, in a specific window given that the participant was in state j during that window. Here, we modeled R(y|j) as a Gaussian random vector with an expectation vector given by μ_j_ and a covariance matrix given by Σ_j_.

**Figure 1:**
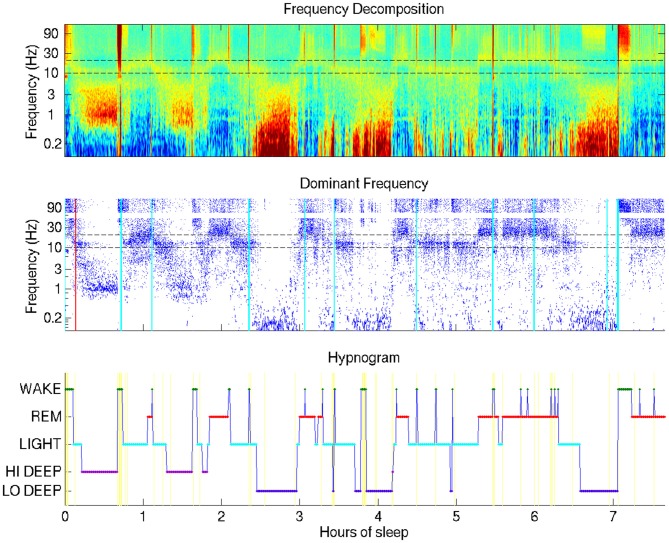
**Typical sleep report showing whole-night sleep electroencephalogram (EEG) from a single forehead electrode (referenced to mastoid).** This night shows Hi Deep sleep in the first and second cycles and Lo Deep sleep in the third and fourth cycles (with a final Lo Deep period before 7 h). Brief power in the high frequency range is indicated with cyan vertical lines—moments of likely micro-arousals when electrodes were moved or high frequency brain activity was temporarily active, or both.

All of the parameters, namely Q and (μ_j_, Σ_j_) j = 1:5 were estimated via the EM algorithm (Rabiner and Juang, 1986) where the initial condition for the diagonal entries of Q (pertaining to the probability of staying in the same stage as the previous interval) were set at 0.95. All off-diagonal entries in the initial condition were set at 0.0125. The estimated parameters were given by Q* and (μ*, Σ*). Given these estimates, the Viterbi algorithm was used to find the maximum a posteriori estimate of the sequence of states. This was used as our estimate of the 5-stage hypnogram. See Figures 1, 3 for examples.

## Results

The visualization technique employed in this study provides a means by which researchers or clinicians, and even patients, can assess an entire night of sleep at a glance. Figure 1 shows the basic view that includes a time-frequency decomposition of a single channel of forehead EEG (baselined by mean spectrum), a dot display highlighting the frequency with the highest relative power at each time point, and a hypnogram generated by the automated HMM algorithm, providing basic guidance for the user to interpret the EEG spectral data plots above it.

The most surprising novel observation that became immediately clear from the whole-night sleep display was that “Deep” sleep occupied two distinct frequency bands, which generally occurred during separate cycles in the night. The difference between what this report coins “Lo Deep” and “Hi Deep” are clearly depicted in Figure 1. Lo Deep has dominant power between 0.1–1 Hz; Hi Deep has very little power in the lowest frequency range and dominant power between 1 Hz and 3 Hz. It is worth noting that, if Figure 1 was calculated and plotted down to 1 Hz only, Hi Deep and Lo Deep would look identical. Therefore, this finding is both a matter of optimal visualization and lowering the frequency range below what is typically considered.

Table 1 summarizes the average hours and percentage of the night spent in each stage of sleep and wake after sleep onset, according to the automatic sleep scoring algorithm. A total of 126 nights from 51 participants were used for these sleep stage quantifications.

**Table 1:** **Duration and percentage of night in each sleep stage**.

	Awake	Light	Hi Deep	Lo Deep	REM
**Number of hours**	0.5 ± 0.4	1.9 ± 0.6	1.2 ± 0.6	1.5 ± 0.7	2.3 ± 0.8
**Percentage of night**	12.8 ± 8.9	26.0 ± 6.4	16.0 ± 7.6	21.2 ± 10.7	30.4 ± 6.9

*Values are means ± standard deviations across participants, derived from the Hidden Markov Model/estimation-maximization automatic sleep scoring algorithm. “Awake” is after sleep onset and does not include time to fall asleep at the beginning of the night. REM, rapid eye movement*.

**Figure 2:**
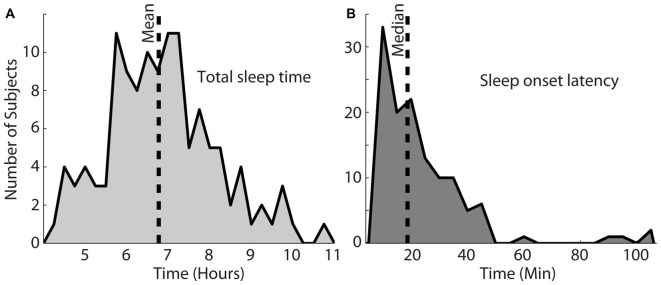
**Total sleep time for this population, shown in (A)** varied from 4.2 h to 10.7 h (mean = 6.8 h). **(B)** Shows the sleep onset latency, which varied between 8.6 min and 214.3 min with a median of 18.9 min. The vertical dotted lines in each plot indicate the mean or median of each distribution.

Figure 2A shows the total sleep time for this particular set of participants. The shortest and longest sleep time was 4.2 h and 10.7 h, respectively, with a mean of 6.8 h. Figure 2B shows the sleep onset latency in minutes, which varied from 8.6 min to 214.3 min (*M* = 18.9 min). The longest two sleep onsets of 167 and 214 min were both achieved by one subject and not shown on the histogram. Most nights (64%), participants fell asleep within 25 min (Figure 2B).

**Figure 3:**
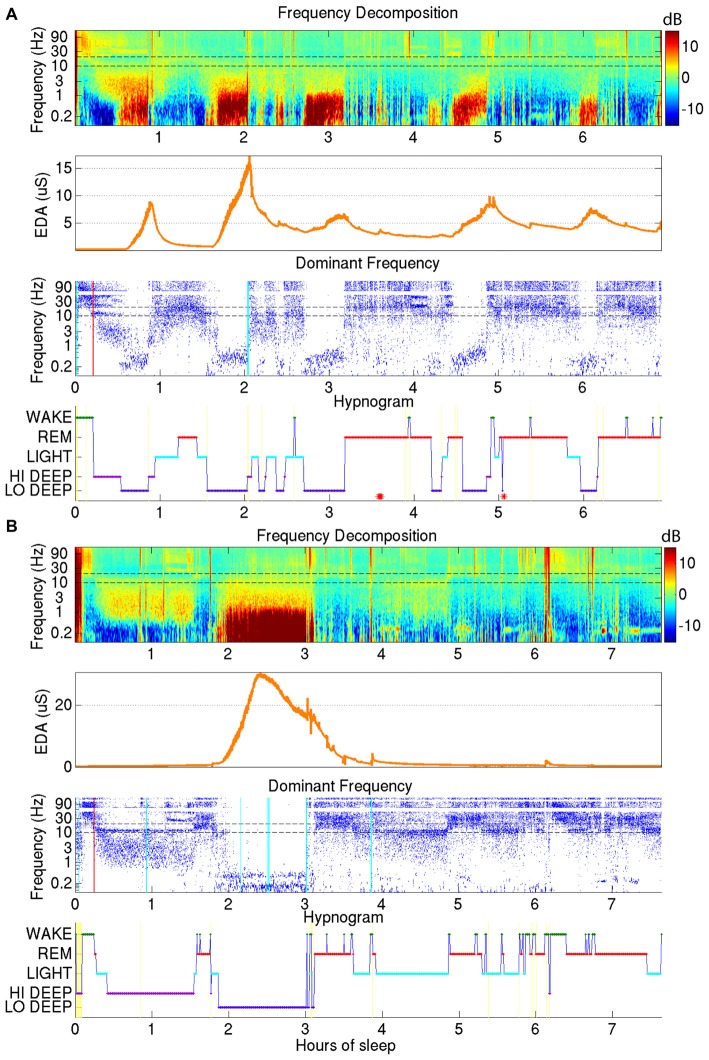
**(A)** Demonstrates how electrodermal activity (EDA; orange trace, second plot down) tends to peak during Lo Deep sleep, usually near the end of the stage. EDA magnitude can vary across cycles. **(B)** Shows another example of EDA increasing during Lo Deep but not during Hi Deep sleep. In this case, EDA peaked before the end of Lo Deep but still showed an accelerated decline at the offset of Lo Deep sleep.

EDA was recorded on the internal surface of the wrist using a watch-like wristband that was approximately synchronized with the EEG from the forehead. Two examples of sleep reports that include EDA data are shown in Figure 3. As can be seen in the second panel down in Figures 3A,B, the EDA measurement was closely related to Lo Deep sleep stages, though the magnitude of the EDA increase was inconsistent between different cycles. EDA appears to start rising close to the beginning of Lo Deep stages, peaks at some point during Lo Deep (Figure 3B), or more commonly rises until the last moment of the stage and then quickly falls toward baseline after the Lo Deep stage ends (Figure 3A). Importantly, it rarely rises during Hi Deep sleep (Figure 3B).

**Figure 4:**
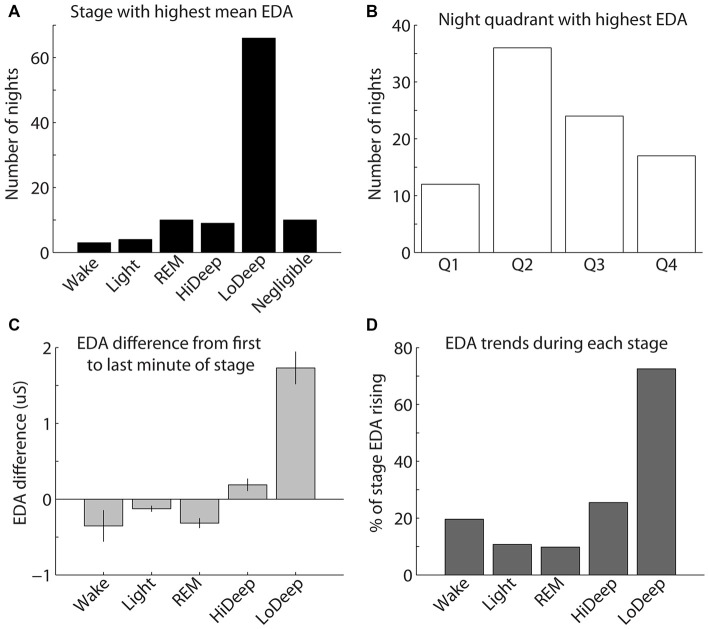
**(A)** The highest mean EDA was usually recorded in a Lo Deep stage of sleep. **(B)** The highest mean EDA was most commonly recorded in the second quarter of the night, although the highest mean EDA could occur during any quadrant of the night. **(C)** EDA tends to be higher during the last minute of Lo Deep stages compared with the first minute of the stage, meaning that EDA tends to rise during Lo Deep (error bars show the standard error of the mean). **(D)** Percentage of time in each stage that EDA was rising rather than falling (using the derivative of EDA measurement). This pattern again shows that EDA tends to increase most dramatically during Lo Deep sleep.

EDA measurement was only successful in a subset of the total participants recorded; therefore, the following results reflect EDA data for 97 nights from 45 participants. To quantify the above EDA observations, the mean EDA during each sleep stage was divided by the mean EDA from all other stages combined. The calculation was restricted to participants with mean EDA in any stage above 0.25 μS because below this level the ratio of means can seem significant with no peak in the EDA measurement. The results show a consistent tendency for EDA to increase most during Lo Deep than in any other sleep stage (Figure 4A). In 10 nights (5 participants), EDA did not reach the threshold value of 0.25 μS for any sleep stage. Of these five participants, three showed minimal and relatively low spectral power Lo Deep sleep that was often mixed with Hi Deep frequency range power. One subject showed clear Lo Deep sleep during three nights, but the EDA was low and erratic, possibly indicating a faulty connection to the skin. The last subject showed clear Lo Deep sleep stages, and EDA increases roughly correlated with Lo Deep stages; however, the magnitude of EDA was so low that it did not reach the threshold value of 0.25 μS over the course of the night.

The highest mean EDA could be expressed in any quarter of the night after sleep onset across participants and recordings (Figure 4B). For the 40 participants (87 nights) with clear peaks in EDA during the night, most nights showed the maximum mean EDA during the second and third quarters of the night (35% and 22%, respectively). Relatively fewer nights showed maximum mean EDA during the first and fourth quarters of the night (11% and 17%, respectively). Nights with negligible EDA throughout the night were excluded from this plot.

As shown in Figure 3, EDA tended to rise slowly during Lo Deep and reach a maximum near the end of the stage. To quantify this, mean EDA from stages lasting ≥8 min, with only one or two sample excursions to other stages tolerated, were taken from the first and last minute to show the difference from beginning to end of each sleep stage category (Figure 4C). Stages throughout the night were used for this analysis, even though EDA can vary significantly from cycle to cycle. All nights from all participants were included in this analysis to portray the average activity for each stage regardless of the overall pattern. Our analysis of variance (ANOVA) results demonstrate that Lo Deep had a significantly higher mean difference from beginning to end of stage compared with all other stages (*P* < 0.0001, *F* = 55.8, effect size [difference/standard deviation]: 0.58 (Wake), 0.65 (Light), 0.71 (REM), 0.4 (Hi Deep)). Of the sleep stages, REM appeared to show the most negative difference, indicating that it generally starts higher than it ends, though it only differed significantly from Hi and Lo Deep sleep stages (*P* < 0.0001, *F* = 55.8, effect size: 0.44 (Hi Deep), 0.71 (Lo Deep)).

To further quantify this EDA behavior, the derivative of the EDA signal was calculated for all nights and expressed as the percentage of time that EDA was rising (positive derivative above 0.0001). Using this metric, Lo Deep sleep again appeared to spend the most time with increasing EDA (72%), while all other stages showed rising EDA 25% of the time or less (Figure 4D).

## Discussion

In this report, we have presented a method for whole-night visualization of sleep EEG from a single channel that vividly highlights the various dominant frequencies characterizing different sleep stages throughout the night. The advantage to this display is that it goes beyond the hypnogram to offer researchers and clinicians an insight into patient sleep EEG that is more comprehensive than the current sleep staging categories. Being able to view the actual EEG data in spectral form can help confirm the automatic sleep scoring, and perhaps convey nuanced aspects of the data that may be related to general sleep quality, although this idea must be further investigated. Our results suggest that the spectrogram and dominant frequency displays can provide complementary descriptive information that can supplement what is provided in the conventional hypnogram. At a glance, one will be able to see if there are discernable cycles, their approximate length, and what sleep stages were reached or missed. Moreover, these descriptive tools will elucidate the number and durations of awakenings, as well as the relative power of dominant and possibly secondary frequencies in each sleep stage.

In addition, we introduced in this report a novel differentiation of deep sleep into Hi and Lo Deep sleep, as defined by maximal power in the 1–3 Hz and <1 Hz range, respectively. Slow wave sleep frequencies are referred to as delta (1–4 Hz) or slow oscillations (<1 Hz) and are suggested to reflect thalamo-cortical and intra-cortical processes, respectively (Steriade et al., 1993). Slow oscillations appear to synchronize faster oscillations in the delta, spindle and even gamma ranges (Steriade, 2000). It is important to point out that Lo Deep sleep, as it is denoted in this report, is not devoid of delta power, rather oscillations below 1 Hz are stronger than delta. That is to say if the spectral power were only calculated down to 1 Hz, Hi and Lo Deep sleep would appear identical. The presence of slow oscillations is likely to initiate a unique network synchrony that presumably performs necessary functions during sleep. For example, slow oscillations may facilitate synaptic pruning (Tononi and Cirelli, 2006), memory consolidation (Stickgold, 2005) and/or neuroplasticity (Dickson, 2010). Thus, the balance of time spent in Hi and Lo Deep sleep may indicate the extent to which various critical neural processes are fulfilled during sleep. The absence, especially, of Lo Deep sleep may therefore hint at serious problems with brain health that could be detected with this simple and inexpensive technique.

The Hi/Lo Deep sleep distinction is easily observed visually in the spectrogram and dominant frequency displays, but it is also usually clear because EDA increases most often during the Lo Deep stage and rarely with the Hi Deep stage—suggesting a physiological difference between the two stages. Until now, Hi and Lo Deep sleep would have been considered the same stage by conventional scoring procedures, which may have confounded earlier studies looking for a consistent association of EDA with “deep” sleep. Despite this, most EDA studies from the 1960s until now have reported the strongest association of EDA with “deep” sleep (Sano et al., 2014), confirming our finding that EDA was not usually strongest during REM or Light sleep.

In the present study, participants showed variable patterns of Hi/Lo Deep sleep and corresponding EDA. Some participants had a full one or two cycles at the beginning of the night that included purely Hi Deep. These participants usually expressed Lo Deep in subsequent cycles and, likewise, their EDA did not rise until the third and later cycles along with the emergence of Lo Deep sleep. Other participants immediately entered Lo Deep during the first cycle and showed the corresponding EDA at the same time. In addition, we also saw instances of EDA and Lo Deep sleep emerging in separable parts of the night, to include once or twice at the beginning of the night and again during the last full cycle of the night. This variance in Hi/Lo Deep patterns within and across participants may partially account for inconsistent findings in the literature reporting that EDA was not always associated with slow-wave sleep in some cycles—thus leading to the conclusion that EDA is somewhat loosely associated with slow-wave sleep (Sano et al., 2014). While the most common sleep stage to show maximal EDA was Lo Deep, we observed that EDA is not equally strong across all cycles containing Lo Deep in a single night, which could also account for apparently skewed correlations between EDA and Lo Deep.

To date, no theories have been proposed to explain the purpose or mechanism of the increase in EDA during slow-wave sleep. During waking conditions, EDA is usually considered a marker of sympathetic nervous system activation as it is often triggered by emotional stimuli while awake (Sequeira et al., 2009). However, non-REM sleep is associated with relatively more parasympathetic and less sympathetic activity in general (Burgess et al., 1997). Indeed, direct recording of sympathetic nerve activity during sleep at the peroneal nerve near the knee shows that nerve activity is mostly silent during deep sleep and highly active during wake and REM (Somers et al., 1993). Nevertheless, it is evidently possible to activate sweat glands, which are exclusively innervated by the sympathetic nervous system, in the absence of other typical sympathetic functions such as increased heart rate and bronchodilation. One explanation for the increase in EDA may be thermoregulation, which is active during deep sleep but not during REM (Carskadon and Dement, 2011).

In rare instances, EDA was expressed in relatively high magnitude in other sleep stages, especially during REM, which may reflect an actual emotional response to a dream, as would be the interpretation while awake. Our results also show it is possible to express Lo Deep sleep EEG activity without measurable EDA changes, meaning that EDA is not directly triggered by Lo Deep sleep production but simply associated with it by unknown mechanisms.

Interestingly, the hormone renin also increases during slow-wave sleep and decreases during REM (Brandenberger et al., 1990). Renin is a factor involved with regulation of extracellular volume of blood, lymph, and interstitial fluid, and is also a target of sympathetic nervous system activation. As with EDA, the purpose and mechanism of this nocturnal pattern is unknown, but appears to be adrenergically mediated since it can be blocked by atenolol (Brandenberger et al., 1990). Possibly, EDA and renin release work together by opening sweat glands in case interstitial fluid levels require regulation through sweating. However, this hypothesis would require explicit testing.

Slow-wave sleep has recently been associated with an actual shrinkage of brain size in rats to allow more cerebrospinal fluid to flow through the brain and clear accumulated debris from the intercellular space (Xie et al., 2013). While it is difficult to say if slow-wave sleep is comparable in rats and humans, including whether rats express both Hi and Lo Deep sleep, it would be extremely valuable to know if and in what stage humans show the same shrinkage phenomenon. Potentially, lack of either Hi or Lo Deep sleep could be correlated with insufficient brain shrinkage and therefore inadequate clearance of proteins that could promote various neurodegenerative diseases. With the low-cost sleep monitoring and scoring presented in this report, patients could be non-invasively identified as having potentially poor cerebrospinal fluid flux well before any irreversible damage occurred.

Finally, growth hormone (GH) is another factor that is released in conjunction with onset of slow-wave sleep (Holl et al., 1991). Responsible for muscle and bone growth, among other functions, GH and slow-wave sleep are reduced in normal aging, acute depression, and after administration of corticotropin-releasing hormone (Steiger and Holsboer, 1997). Knowing whether this is related to Hi, Lo, or all Deep sleep and whether the magnitude of deep sleep’s spectral power relates to the amount of GH release would enhance the informative value of detecting the amount of Hi vs. Lo Deep sleep.

Thus, a constellation of physiological events occur during slow-wave sleep, and many events are still unknown. That these factors may, like EDA, be associated with only Lo Deep sleep suggests that monitoring patients for the ratio of Hi to Lo Deep sleep could expose problems with sleep-related bodily functions that would not otherwise be obvious from routine sleep assessments. It may be that sleep complaints, in conditions ranging from PTSD to advanced age, are due to skewed Hi/Lo Deep sleep ratios. Additionally, the influence of pharmacological medication on Hi vs. Lo Deep sleep ratios should also be investigated since they may have consequences on sleep quality that are as yet undetected.

The sleep stages used in the present study contain both Hi and Lo Deep categories and thus cannot be compared with conventional scoring. However, the variance in values seems to expose interesting differences between spectral and temporal classification that should be highlighted. In the present study, the percentage of the night spent in REM was similar to commonly reported values (28.6% vs. 20%–25%; Carskadon and Dement, 2011). In non-REM sleep, the values for combined stages 3 and 4 are usually considered to be in the range of 13%–23%, while stage 2 sleep is said to consume approximately 45%–55% of the night. These values contrast somewhat with the results presented here, which showed Hi and Lo Deep sleep (should approximate stages 3 and 4) persisting for 53.2% of the night, on average, and Light sleep (should be similar to stage 2) for 24.8%. This apparent reversal of time spent in Light vs. Deep sleep might be explained by the 12- to 14-Hz spindle activity (i.e., marker of Light sleep) which is clearly strong during concurrent slow waves. This observation implies that individual 30-s stretches are likely to exist that contain <20% high-amplitude slow waves and >0.5 s of spindle activity that would visually be scored as stage 2 (Silber et al., 2007). Using the spectral method presented here, the amplitude of frequency power is the more important factor. Thus, large amplitude slow-waves in only 20% of the epoch could appear to the algorithm as Hi or Lo Deep sleep even in the presence of frequent spindle activity. Also, slow waves are known to be stronger at frontal electrodes (Kurth et al., 2010), but prior to new guidelines in 2007, traditional scoring used central derivations instead of the current standard which uses frontal electrodes for visual scoring (Silber et al., 2007). This means that even visual scoring of frontal electrode data is potentially incomparable with commonly accepted values since the decision between stage 2 and slow-wave sleep depends partly on the amplitude of delta activity. However, the main reason for the difference between values in the current results and commonly reported values is likely that the scoring rules in this report did not attempt to mimic standard visual scoring rules. Rather, our algorithm uses the whole-night spectral macrostructure to make data-driven differentiations between sleep stages based on spectral rather than temporal dynamics. Importantly, both are perfectly valid approaches to data interpretation; the current one simply has the advantage of being far quicker and providing more information to the end user through the spectrogram and hypnogram.

The idea of whole-night sleep visualization using the power spectrogram has been previously proposed (Kokkinos et al., 2009; Koupparis et al., 2014). In these reports, the authors point out several of the same findings, specifically that imaging whole-night sleep can provide a quick and efficient overview of the whole night and certain spectral bands are highly correlated with certain sleep stages (e.g., spindles with light sleep and slow waves with deep sleep). However, these reports did not indicate the presence of two distinct frequency bands in deep sleep. There are several possible reasons for this. First, the Cz derivation used or the roll-off from the 0.05-Hz high-pass filter may have obscured some of the Lo Deep sleep power. Alternatively, the limitations of their display may have prevented visual detection of the phenomenon, specifically the use of linear-spaced frequencies in the spectrogram that allows very little space for the entire low frequency range to be viewed. The current report improves upon this approach by employing log-scale frequency spacing that clearly distinguishes between Lo Deep and Hi Deep. In addition, the color scaling and smoothing, along with the dominant frequency display, provide a more informative depiction of sleep EEG’s frequency characteristics.

Our approach for automated sleep staging consists of an HMM and performs maximum likelihood estimation on the parameters (via the EM algorithm) and maximum *a posteriori* estimation of the most likely hypnogram (via the Viterbi algorithm). As a method for state classification, the HMM/EM/Viterbi framework provides an unsupervised approach for objectively identifying the hidden states of sleep from continuous EEG observations. In general, HMMs are widely studied and employed in fields such as speech recognition (Rabiner, 1989) and gene editing (Eddy, 2004), hence they are well characterized and amenable for use in the spectral analysis of sleep EEG described here. In contrast to other sleep scoring schemes employed in the literature (e.g., support vector machines, neural networks, decision trees), HMMs account for inherent temporal structure in an observed signal; this signal allows for a statistical analysis that ultimately constrains classification of hidden sleep states in a manner commensurate with physiological manifestations and transitions during sleep.

The current study utilized a 2-channel mobile EEG device that participants were able to apply, and sleep with, in the comfort of their own homes. This aspect of our study means that we were able to acquire data that is closer to natural sleep patterns; sleeping in one’s own bed is far more comfortable than sleeping in a foreign bed with technicians looking on. Furthermore, with repeated in-home EEG recordings being relatively inexpensive, these devices allow for more accommodation nights so that typical patterns for each patient or participant can be more accurately determined. These devices are becoming more available as applications for EEG expand and methods like the one presented here provide a means for clinicians or researchers to quickly and accurately analyze sleep recordings.

Typically reported sleep onset latency ranges from about 15–20 min, on average (Ohayon et al., 2004), which was slightly lower than the average in the present report. However, the majority (64%) of the participants fell asleep in less than 25 min. The current values are therefore similar to reported values in most cases, demonstrating that most of the participants did not experience problems falling asleep with the sleep recording device on their heads.

In summary, we have presented evidence for a new categorization of deep sleep that separates slow-wave sleep according to the dominant frequency and coincident EDA patterns. Finally, this report demonstrates the feasibility of inexpensive, high quality, in-home sleep monitoring that can quickly assess sleep architecture and, potentially, overall sleep quality.

## Ethics Statement

This study was approved by the Institutional Review Board of the Naval Health Research Center (IRB Protocol NHRC.2016.0047) in San Diego. Participants were informed of all study procedures and all aspects of consent form were clearly described to them. They were given time to review the document on their own. Then they were given the opportunity to sign the consent form and participate in the study, or choose not to participate, upon full understanding of the procedures and compensation. No special populations were used in this study.

## Author Contributions

JAO: secured the funding, conducted subject recruitment and data collection, devised spectral decomposition method, including visualization and spectral bands of interest, and wrote the manuscript; DYK and TPC: helped create the automatic scoring algorithm and contributed to manuscript editing.

## Disclaimer

The views expressed in this article are those of the authors and do not necessarily reflect the official policy or position of the Department of the Navy, Department of the Army, Department of the Air Force, Department of Veterans Affairs, Department of Defense, or the US Government. Approved for public release; distribution is unlimited. US Government Work (17 USC §105). Not copyrighted in the United States.

## Conflict of Interest Statement

The authors declare that the research was conducted in the absence of any commercial or financial relationships that could be construed as a potential conflict of interest.

## References

[B1] Baust W. Bohnert B. (1969). The regulation of heart rate during sleep. Exp. Brain Res. 7, 169–180. doi: 10.1007/bf00235442 5799433 10.1007/BF00235442

[B2] Berthomier C. Drouot X. Herman-Stoïca M. Berthomier P. Prado J. Bokar-Thire D.. (2007). Automatic analysis of single-channel sleep EEG: validation in healthy individuals. Sleep 30, 1587–1595. 18041491 10.1093/sleep/30.11.1587 PMC2082104

[B3] Brandenberger G. Krauth M. O. Ehrhart J. Libert J. P. Simon C. Follenius M. (1990). Modulation of episodic renin release during sleep in humans. Hypertension 15, 370–375. doi: 10.1161/01.hyp.15.4.370 2180817 10.1161/01.hyp.15.4.370

[B4] Burgess H. J. Trinder J. Kim Y. Luke D. (1997). Sleep and circadian influences on cardiac autonomic nervous system activity. Am. J. Physiol. 273, H1761–H1768. 9362241 10.1152/ajpheart.1997.273.4.H1761

[B5] Carskadon M. A. Dement W. C. (2011). “Monitoring and staging human sleep,” in Principles and Practice of Sleep Medicine, 5th Edn. eds Kryger M. H. Roth T. Dement W. C. (St. Louis: Elsevier Saunders), 16–26.

[B6] Danker-Hopfe H. Anderer P. Zeitlhofer J. Boeck M. Dorn H. Gruber G.. (2009). Interrater reliability for sleep scoring according to the Rechtschaffen & Kales and the new AASM standard. J. Sleep Res. 18, 74–84. doi: 10.1111/j.1365-2869.2008.00700.x 19250176 10.1111/j.1365-2869.2008.00700.x

[B7] Dawson M. E. Schell A. M. Filion D. L. (2007). “The electrodermal system,” in Handbook of Psychophysiology, eds Cacioppo J. T. Tassinary L. G. Berntson G. G (Cambridge: Cambridge University Press), 159–181.

[B8] Dickson C. T. (2010). Ups and downs in the hippocampus: the influence of oscillatory sleep states on “neuroplasticity” at different time scales. Behav. Brain Res. 214, 35–41. doi: 10.1016/j.bbr.2010.04.002 20394778 10.1016/j.bbr.2010.04.002

[B9] Eddy S. R. (2004). What is a hidden Markov model? Nat. Biotechnol. 22, 1315–1316. doi: 10.1038/nbt1004-1315 15470472 10.1038/nbt1004-1315

[B10] Flexer A. Dorffner G. Sykacekand P. Rezek I. (2002). An automatic, continuous and probabilistic sleep stager based on a hidden markov model. Appl. Artif. Intell. 16, 199–207. doi: 10.1080/088395102753559271

[B11] Flexer A. Gruber G. Dorffner G. (2005). A reliable probabilistic sleep stager based on a single EEG signal. Artif. Intell. Med. 33, 199–207. doi: 10.1016/j.artmed.2004.04.004 15811785 10.1016/j.artmed.2004.04.004

[B12] Holl R. W. Hartman M. L. Veldhuis J. D. Taylor W. M. Thorner M. O. (1991). Thirty-second sampling of plasma growth hormone in man: correlation with sleep stages. J. Clin. Endocrinol. Metab. 72, 854–861. doi: 10.1210/jcem-72-4-854 2005213 10.1210/jcem-72-4-854

[B13] Hori T. Miyasita A. Niimi Y. (1970). Skin potential activities and their regional differences during normal sleep in humans. Jpn. J. Physiol. 20, 657–671. doi: 10.2170/jjphysiol.20.657 4324224 10.2170/jjphysiol.20.657

[B14] Johnson L. C. Lubin A. (1966). Spontaneous electrodermal activity during waking and sleeping. Psychophysiology 3, 8–17. doi: 10.1111/j.1469-8986.1966.tb02673.x 5942878 10.1111/j.1469-8986.1966.tb02673.x

[B15] Kokkinos V. Koupparis A. Stavrinou M. L. Kostopoulos G. K. (2009). The hypnospectrogram: an EEG power spectrum based means to concurrently overview the macroscopic and microscopic architecture of human sleep. J. Neurosci. Methods 185, 29–38. doi: 10.1016/j.jneumeth.2009.09.002 19747945 10.1016/j.jneumeth.2009.09.002

[B16] Koupparis A. M. Kokkinos V. Kostopoulos G. K. (2014). Semi-automatic sleep EEG scoring based on the hypnospectrogram. J. Neurosci. Methods 221, 189–195. doi: 10.1016/j.jneumeth.2013.10.010 24459717

[B17] Kurth S. Ringli M. Geiger A. LeBourgeois M. Jenni O. G. Huber R. (2010). Mapping of cortical activity in the first two decades of life: a high-density sleep electroencephalogram study. J. Neurosci. 30, 13211–13219. doi: 10.1523/JNEUROSCI.2532-10.2010 20926647 10.1523/JNEUROSCI.2532-10.2010 PMC3010358

[B18] Liang S.-F. Kuo C.-E. Hu Y.-H. Cheng Y.-S. (2012). A rule-based automatic sleep staging method. J. Neurosci. Methods 205, 169–176. doi: 10.1016/j.jneumeth.2011.12.022 22245090 10.1016/j.jneumeth.2011.12.022

[B19] Ohayon M. M. Carskadon M. A. Guilleminault C. Vitiello M. V. (2004). Meta-analysis of quantitative sleep parameters from childhood to old age in healthy individuals: developing normative sleep values across the human lifespan. Sleep 27, 1255–1273. 15586779 10.1093/sleep/27.7.1255

[B20] Pan S. T. Kuo C. E. Zeng J. H. Liang S. F. (2012). A transition-constrained discrete hidden Markov model for automatic sleep staging. Biomed. Eng. Online 11:52. doi: 10.1186/1475-925x-11-52 22908930 10.1186/1475-925X-11-52 PMC3462123

[B21] Pardey J. Roberts S. Tarassenko L. Stradling J. (1996). A new approach to the analysis of the human sleep/wakefulness continuum. J. Sleep Res. 5, 201–210. doi: 10.1111/j.1365-2869.1996.00201.x 9065871 10.1111/j.1365-2869.1996.00201.x

[B22] Rabiner L. R. (1989). A tutorial on hidden Markov models and selected applications in speech recognition. Proc. IEEE 77, 257–286. doi: 10.1109/5.18626

[B23] Rabiner L. R. Juang B.-H. (1986). An introduction to hidden Markov models. ASSP Mag. IEEE 3, 4–16. doi: 10.1109/MASSP.1986.1165342

[B24] Rechtschaffen A. Kales A. (1969). A manual of standardized terminology, techniques and scoring system for sleep stages of human subjects. Clin. Neurophysiol. 26:644 doi: 10.1016/0013-4694(69)90021-2 10.1046/j.1440-1819.2001.00810.x 11422885

[B25] Sano A. Picard R. W. Stickgold R. (2014). Quantitative analysis of wrist electrodermal activity during sleep. Int. J. Psychophysiol. 94, 382–389. doi: 10.1016/j.ijpsycho.2014.09.011 25286449 10.1016/j.ijpsycho.2014.09.011 PMC4335672

[B26] Sequeira H. Hot P. Silvert L. Delplanque S. (2009). Electrical autonomic correlates of emotion. Int. J. Psychophysiol. 71, 50–56. doi: 10.1016/j.ijpsycho.2008.07.009 18723054 10.1016/j.ijpsycho.2008.07.009

[B27] Silber M. H. Ancoli-Israel S. Bonnet M. H. Chokroverty S. Grigg-Damberger M. M. Hirshkowitz M.. (2007). The visual scoring of sleep in adults. J. Clin. Sleep Med. 3, 121–131. 17557422

[B28] Somers V. K. Dyken M. E. Mark A. L. Abboud F. M. (1993). Sympathetic-nerve activity during sleep in normal subjects. N. Engl. J. Med. 328, 303–307. doi: 10.1056/NEJM199302043280502 8419815 10.1056/NEJM199302043280502

[B29] Sousa T. Cruz A. Khalighi S. Pires G. Nunes U. (2015). A two-step automatic sleep stage classification method with dubious range detection. Comput. Biol. Med. 59, 42–53. doi: 10.1016/j.compbiomed.2015.01.017 25677576 10.1016/j.compbiomed.2015.01.017

[B30] Steiger A. Holsboer F. (1997). Nocturnal secretion of prolactin and cortisol and the sleep EEG in patients with major endogenous depression during an acute episode and after full remission. Psychiatry Res. 72, 81–88. doi: 10.1016/s0165-1781(97)00097-8 9335199 10.1016/s0165-1781(97)00097-8

[B31] Steriade M. (2000). Corticothalamic resonance, states of vigilance and mentation. Neuroscience 101, 243–276. doi: 10.1016/s0306-4522(00)00353-5 11074149 10.1016/s0306-4522(00)00353-5

[B32] Steriade M. Nuñez A. Amzica F. (1993). Intracellular analysis of relations between the slow (<1 Hz) neocortical oscillation and other sleep rhythms of the electroencephalogram. J. Neurosci. 13, 3266–3283. 8340807 10.1523/JNEUROSCI.13-08-03266.1993 PMC6576520

[B33] Stickgold R. (2005). Sleep-dependent memory consolidation. Nature 437, 1272–1278. doi: 10.1038/nature04286 16251952 10.1038/nature04286

[B34] Tononi G. Cirelli C. (2006). Sleep function and synaptic homeostasis. Sleep Med. Rev. 10, 49–62. doi: 10.1016/j.smrv.2005.05.002 16376591 10.1016/j.smrv.2005.05.002

[B35] Xie L. Kang H. Xu Q. Chen M. J. Liao Y. Thiyagarajan M.. (2013). Sleep drives metabolite clearance from the adult brain. Science 342, 373–377. doi: 10.1126/science.1241224 24136970 10.1126/science.1241224 PMC3880190

[B36] Yaghouby F. Sunderam S. (2015). Quasi-supervised scoring of human sleep in polysomnograms using augmented input variables. Comput. Biol. Med. 59, 54–63. doi: 10.1016/j.compbiomed.2015.01.012 25679475 10.1016/j.compbiomed.2015.01.012 PMC4447106

